# Structured light imaging mesoscopy: detection of embedded morphological changes in superficial tissues

**DOI:** 10.1117/1.JBO.30.6.065001

**Published:** 2025-06-18

**Authors:** Mahsa Parsanasab, Aarohi Mahesh Mehendale, Kavon Karrobi, Darren Roblyer, Vasan Venugopalan

**Affiliations:** a University of California, Irvine, Department of Chemical and Biomolecular Engineering, Irvine, California, United States; b University of California, Irvine, Beckman Laser Institute and Medical Clinic, Irvine, California, United States; c Boston University, Department of Biomedical Engineering, Boston, Massachusetts, United States; d Boston University, Department of Electrical and Computer Engineering, Boston, Massachusetts, United States

**Keywords:** subsurface morphological change detection, spatial frequency domain measurement, Monte Carlo simulation, perturbation methods, inverse problems

## Abstract

**Significance:**

Current paradigms for the optical characterization of layered tissues involve explicit consideration of an inverse problem which is often ill-posed and whose resolution may retain significant uncertainty. Here, we present an alternative approach, structured light imaging mesoscopy (SLIM), that leverages the inherent sensitivity of raw spatial frequency domain (SFD) reflectance measurements for the detection of embedded subsurface scattering changes in tissue.

**Aim:**

We identify wavelength–spatial frequency ($\lambda \text{-}f_{x}$) combinations that provide optimal sensitivity of SFD reflectance changes originating from scattering changes in an embedded tissue layer. We specifically consider the effects of scattering changes in the superficial dermis which is a key locus of pathology for diverse skin conditions such as cancer, aging, and scleroderma.

**Approach:**

We used Monte Carlo simulations in a four-layer skin model to analyze the SFD reflectance changes resulting from changes in superficial dermal scattering across wavelength (λ=471 to 851 nm) and spatial frequency ($f_{x}=0$ to 0.5/mm). Within this model, we consider different values for epidermal melanin concentration to simulate variations in skin tone.

**Results:**

Monte Carlo simulations revealed that scattering changes within the superficial dermis produce SFD reflectance changes which are maximized at specific ($\lambda \text{-}f_{x}$) pairs and vary with skin tone. For light skin tones, SFD reflectance changes due to scattering reductions in the superficial dermis are maximized at λ=621  nm and spatial frequency $f_{x}\approx 0.33/\mathrm{mm}$. By contrast, for darker skin tones, maximal SFD reflectance changes occur at wavelengths in the near-infrared (λ≥811  nm) at a spatial frequency of $f_{x}\approx 0.25/\mathrm{mm}$. Interestingly, the change in SFD reflectance produced by such scattering changes is most uniform across all skin tones when using the longest wavelength tested (λ=851  nm) and a spatial frequency of $f_{x}\approx 0.22/\mathrm{mm}$. Taken together, our computational model identifies specific ($\lambda \text{-}f_{x}$) pairs to optimally detect embedded structural alterations in the superficial dermis.

**Conclusions:**

The findings establish the SLIM methodology as a means to detect morphological changes in an embedded subsurface tissue layer by leveraging inherent sensitivities of spatial frequency domain reflectance. This approach promises to enable simplified clinical tracking of subsurface microstructural alterations without the explicit need to consider an inverse problem approach.

## 1 Introduction

This paper represents the first part of a two-part series introducing structured light imaging mesoscopy (SLIM) as a novel optical technique. Here, in part 1, we introduce and motivate the SLIM technique and utilize Monte Carlo (MC) simulations to illustrate how SLIM can be used to detect subsurface scattering changes in the papillary dermis of skin. In part 2, published in* Biophotonics Discovery*,^1^ we demonstrate the application of SLIM for the detection and characterization of scleroderma, an autoimmune disease that alters the optical scattering properties of the skin, particularly in the dermis.

Optical characterization of biological tissues, through the determination of optical properties, has been a cornerstone in biophotonics research, providing valuable insights into tissue structure and function, including layered tissues.^2^^–^^10^ Traditional methods, such as inverse problem approaches, often face challenges due to the complexity of light propagation, especially in superficial layered tissues due to the mesoscopic spatial scales (≲1  mm) and tissue heterogeneity. These challenges can lead to uncertainties and inaccuracies in model-based recovery of optical and physiological properties.^11^^–^^14^ Machine learning techniques, while promising, can lack interpretability and require large training sets, which limits their generality and utility.^15^^–^^20^

Spatial frequency domain imaging (SFDI) is a well-established method that measures the remitted light resulting from structured illumination of tissue at multiple spatial frequencies to derive wide-field images of endogenous biomolecular concentrations and tissue morphology.^21^ Because the optical penetration of light is variable with spatial frequencies on submillimeter and millimeter spatial scales,^22^ measurements of spatial frequency domain (SFD) reflectance at various spatial frequencies and wavelengths can be used to probe different depths in superficial tissues.^22^^,^^23^ However, the sampling of different tissue volumes inherent with the use of multiple spatial frequencies^22^ can complicate the interpretation of SFDI measurements with respect to identifying the region from which changes in SFD reflectance originate and to which optical properties should be assigned. This phenomenon is commonly referred to as the “partial volume effect” and understood in the diffuse optics community to be responsible for inaccuracy when assessing optical properties in heterogeneous tissues.^12^^,^^22^^,^^24^^–^^29^ With the use of appropriate, transport-rigorous SFD computational models, there is a potential opportunity to identify SFD measurement parameters that are maximally sensitive to changes in a subsurface tissue layer on submillimeter spatial scales (mesoscale), thereby enabling a more detailed analysis of tissue structure from SFD measurements.

Here, we introduce SLIM as a technique that leverages the inherent sensitivity of raw SFD reflectance measurements at specific wavelength–spatial frequency ($\lambda \text{-}f_{x}$) pairs to directly detect subsurface scattering changes in a subvolume of interest. This approach, without explicit consideration of an inverse problem, offers a streamlined and efficient method for characterizing superficial tissues. Such a technique could be particularly valuable in clinical settings for monitoring or detection of conditions characterized by alterations in tissue scattering properties, including cancer, aging, and various rheumatic diseases such as scleroderma.

To demonstrate this approach, we examine the structural changes occurring in the superficial dermis, a region where many skin pathologies manifest.^30^^–^^35^ Accurate characterization of this region is essential for early diagnosis and treatment. However, the optical properties of the superficial dermis are difficult to isolate due to its submillimeter thickness (∼300  μm) and its proximity to both the epidermis and the deeper reticular dermis.^36^ In addition, variations in skin pigmentation, hydration levels, and anatomical location can further complicate measurement localization and interpretation of optical signals from this region.^37^ Nevertheless, given the demonstrated sensitivity of SFDI to subtle changes in tissue morphology and hemodynamics,^38^^–^^42^ we aim to demonstrate that SLIM provides a valuable approach to detecting/tracking such changes without explicit consideration of a model-based inverse problem or the use of a classification algorithm.

To demonstrate the SLIM method as an approach to identify measurement parameters that optimally detect structural changes within a specific tissue layer of interest, we perform Monte Carlo simulations in a multi-layer skin model. The simulations are designed to analyze the SFD reflectance changes resulting from variations in superficial dermal scattering coefficients across a range of wavelengths and spatial frequencies. Within this model, we also consider different values for epidermal melanin concentration to simulate the impact of variations in skin tone. These simulations enable the identification of $\lambda \text{-}f_{x}$ pairs that provide a maximal SFD reflectance change resulting from the subsurface structural change.

## 2 Methods

**Fig. 1:**
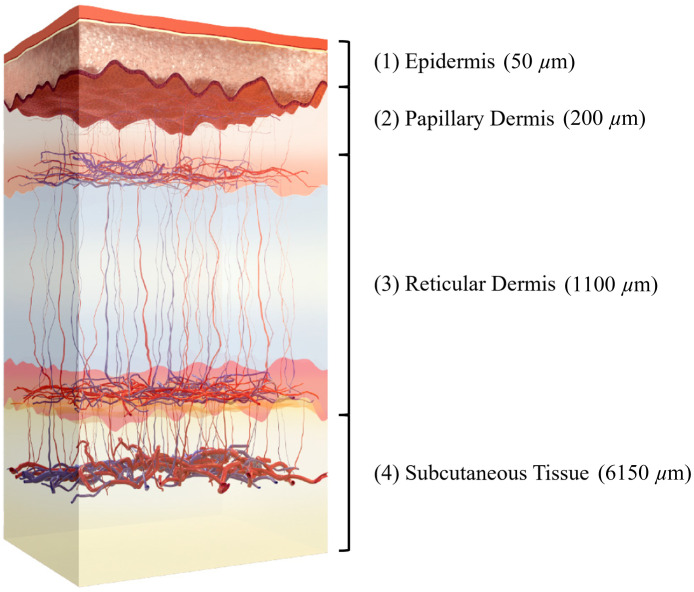
Diagram of the simplified four-layer skin tissue model (adapted from Ref. 43).

In this initial demonstration of SLIM, we utilize a four-layer skin model (Fig. 1) to analyze SFD reflectance measurements in the skin using MC simulations. Our model draws inspiration from the seven-layer skin model developed by Zherebstov and co-workers.^43^ Our four-layer model was designed to provide a framework that captures the essential features of the heterogeneous distributions of primary tissue constituents responsible for SFD reflectance: blood volume fraction, blood oxygenation, water, fat, and tissue scattering within four individual tissue compartments in skin: epidermis, papillary dermis, reticular dermis, and subcutis.^43^^,^^44^

The main parameters of the model are presented in Table 1. Sublayer thicknesses were based on the models presented in Refs. 43, 45, and 46, and subcutaneous tissue scattering coefficients were calculated based on data from Fishkin and co-workers.^47^ The absorption coefficient in dermal layers was calculated using Eq. (1)^43^ and accounted for the layer-specific blood volume fraction, oxygen saturation, water, and lipid content. These specifications are equivalent to the weighted average values based on the thicknesses in the seven-layer model of Zherebstov and co-workers^43^

$$\mu_{a}(\lambda )=2.303\times 10^{-6}(C_{\mathrm{HbO}}e_{\mathrm{HbO}}+C_{\mathrm{HbR}}e_{\mathrm{HbR}})+C_{\mathrm{water}}\mu_{a}^{\mathrm{water}}(\lambda )+C_{\mathrm{lipid}}\mu_{a}^{\mathrm{lipid}}(\lambda ).$$ (1)

In Eq. (1), depending on the layer of interest (epidermis, papillary dermis, reticular dermis, or subcutis), $C_{i}$ and $e_{i}$ represent the concentration and extinction coefficient of the constitutive elements: oxygenated hemoglobin (HbO), reduced hemoglobin (HbR), water, and lipid. These are shown in Table 1.

**Table 1:** Parameters used for the assessment of the absorption coefficients of the multi-layer skin model assuming 15-g/dL hemoglobin level and 80% blood oxygenation.^8^

Layer	Thickness (μm)	Blood volume fraction (%)	Oxygenated blood, $C_{\text{HbO}}$ (μM)	Deoxygenated blood, $C_{\text{HbR}}$ (μM)	Water, $C_{\text{water}}$ (%)	Lipid, $C_{\text{lipid}}$ (%)
Epidermis	50	0	0	0	17	10
Papillary dermis	200	2	37.21	9.30	70	5
Reticular dermis	1100	2	37.21	9.30	70	5
Subcutaneous tissue	6150	7	130.2	32.5	70	23

The absorption due to epidermal melanin content was approximated using Eq. (2) in which $f_{v}$ represents the melanosome volume fraction.^48^ As the density of melanin content within melanosomes is variable and uncertain,^48^ we will simply refer to $f_{v}$ as the epidermal melanin content

$$\mu_{a}^{\mathrm{mel}}(\lambda )\approx \;1.7\times 10^{11}\;f_{v}\lambda^{-3.48}.$$ (2)

The scattering coefficients of the epidermis [Eq. (3)] and both the papillary and reticular dermis [Eq. (4)] were approximated based on the combination of Mie and Rayleigh scattering^43^

$$\mu_{s}^{\prime \mathrm{epidermis}}(\lambda )=1.08\times 10^{7}\lambda^{-2.364}+13.571\lambda^{-0.267},$$ (3)

$$\mu_{s}^{\prime \mathrm{dermis}}(\lambda )=1.19\times 10^{7}\lambda^{-2.427}+7.15\lambda^{-0.258}.$$ (4)

To model the light transport in the multilayer skin model, we used the open-source Monte Carlo Command Line (MCCL) software package^49^ to perform separate conventional Monte Carlo simulations for each of the eight wavelengths used on the commercially available Modulim SFDI system (Reflect RS, Modulim Inc., Irvine, California, United States): λ=471, 526, 591, 621, 691, 731, 811, and 851 nm. For each wavelength, we simulated the propagation of $10^{8}$ photons within our skin model using the wavelength-specific optical properties and determined the SFD reflectance for spatial frequencies in the range of 0 to 1/mm with 0.01/mm increments. The refractive index (n) and scattering anisotropy factor (g) for the epidermis, papillary dermis, reticular dermis, and subcutaneous tissue were set to n/g 1.37/0.8, 1.4/0.8, 1.4/0.8, and 1.44/0.75, respectively.^41^ SFD reflectance estimates were determined using the modified shortcut method developed by Gardner and Venugopalan.^50^ Using this method, the Monte Carlo simulations are performed directly in the spatial frequency domain. This enables the MC simulation to directly determine the weight of each detected photon at each spatial frequency of interest. Such tallies are not subject to the inaccuracies that can result from the determination of SFD reflectance through the use of discrete Fourier/Hankel transforms of spatially resolved reflectance data.^22^

Two sets of conventional Monte Carlo simulations were conducted at each wavelength representing a baseline scenario and a second “perturbed” scenario where the scattering coefficient was reduced by 50% in the papillary dermis layer alone. The perturbed scenario is meant to simulate degeneration of the basement membrane and/or papillary dermis ultrastructure, which is seen in conditions such as aging, skin sclerosis, and invasive basal and squamous cell carcinomas.^51^^–^^55^ For the baseline simulations, we employed conventional Monte Carlo simulation based on the parameters in Table 1 and Eqs. (1)–(4). The detailed optical properties used in the simulations are given in Tables S1–S3 in the Supplementary Material. To simulate SFD reflectance variations for different epidermal melanin concentrations, we utilized perturbation Monte Carlo simulations to provide accurate results with significantly reduced computational cost.^8^^,^^56^^,^^57^

## 3 Results

**Fig. 2:**
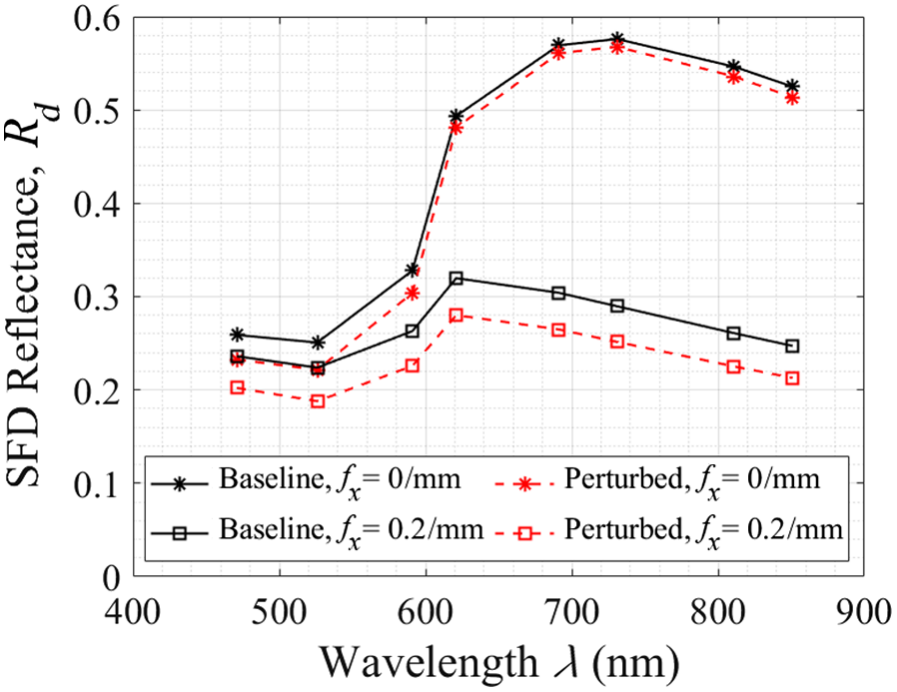
SFD reflectance versus wavelength for baseline (solid black lines) and negative scattering perturbation (dashed red lines) at $f_{x}=0/\mathrm{mm}$ (*) and $f_{x}=\;0.2/\mathrm{mm}$ (▫) with 2% epidermal melanin concentration.

Figure 2 provides the MC simulation results of SFD reflectance spectra at spatial frequencies of $f_{x}=0$ and 0.2/mm in our skin model with 2% epidermal melanin content. For both spatial frequencies, we observe relatively low reflectance values for wavelengths λ≲600  nm with a rapid increase in the SFD reflectance from λ=591 to 691 nm corresponding to the significant drop in hemoglobin absorption in this spectral region. This is followed by a modest plateau in the reflectance for λ≳700  nm. The increase in SFD reflectance is most pronounced at $f_{x}=0/\mathrm{mm}$, i.e., the increase in reflectance diminishes with increasing $f_{x}$. Moreover, due to the lower optical absorption for λ≳700  nm, changes in spatial frequency have a greater effect on the reflectance at these longer wavelengths.

When examining the change in the SFD reflectance from the baseline cases (solid black lines) to the perturbed case (dashed red lines) where the scattering in the papillary dermis is reduced by 50%, we observe a reduction in SFD reflectance across all wavelengths. Interestingly, for uniform/DC illumination ($f_{x}=0/\mathrm{mm}$), we observe the most significant reductions in reflectance for λ≲600  nm and minimal reductions at longer wavelengths. By contrast, for higher spatial frequencies (e.g., $f_{x}=0.2/\mathrm{mm}$), substantive reflectance reductions are seen across all wavelengths.

**Fig. 3:**
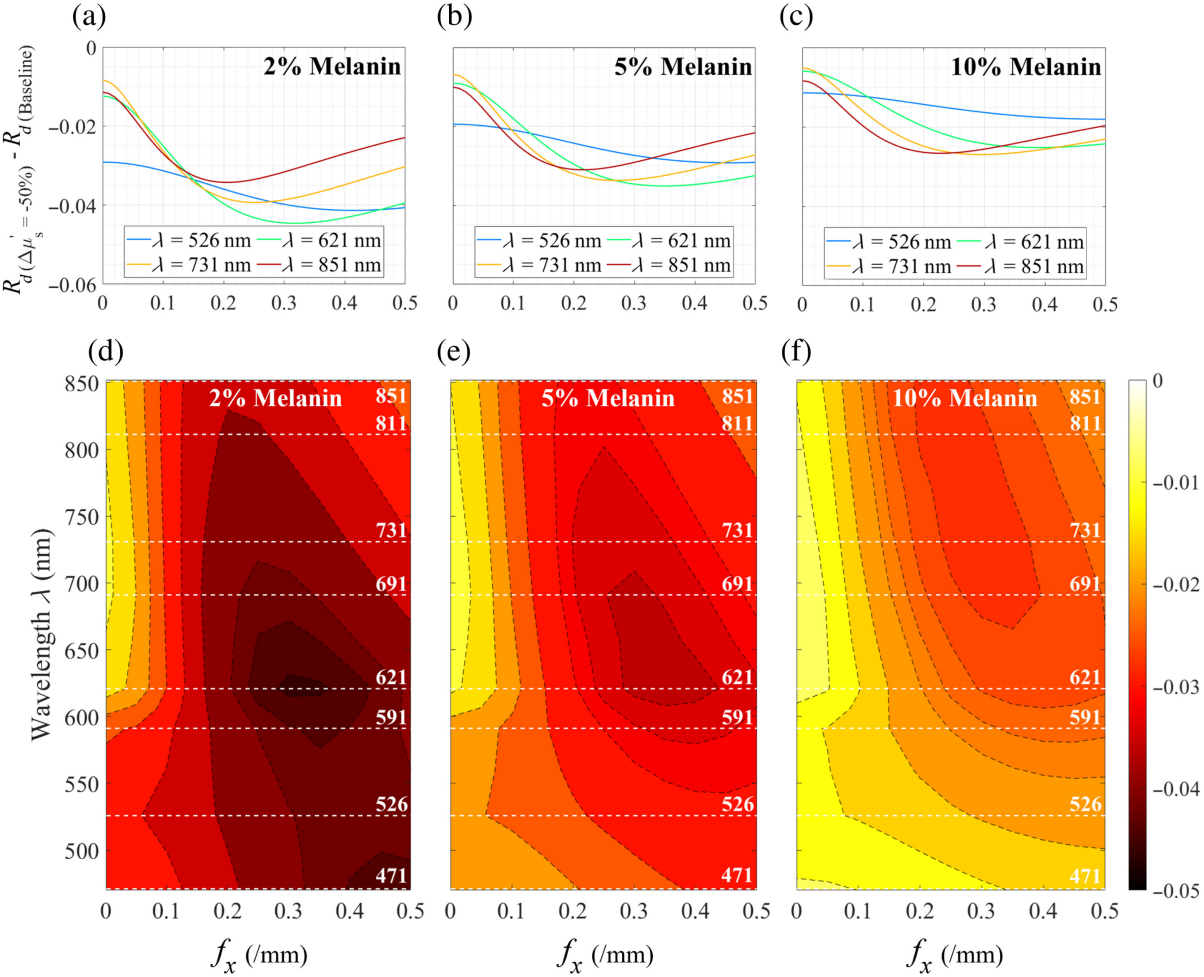
SFD reflectance change $\Delta R_{d}$ versus spatial frequency $f_{x}$ at individual wavelengths for epidermal melanin concentrations of (a) 2%, (b) 5%, and (c) 10%. Contour plots showing the variation of SFD reflectance, $\Delta R_{d}$, with $f_{x}$ and λ for epidermal melanin concentrations of (d) 2%, (e) 5%, and (f) 10%.

In Fig. 3 we present the change in SFD reflectance between the baseline and perturbed cases versus spatial frequency for four select wavelengths and epidermal melanin concentrations of 2%, 5%, and 10%. These epidermal melanin concentrations were chosen to represent light, medium, and dark skin tones.^46^ As depicted in Fig. 3(a), the magnitude of the reflectance changes at λ=526  nm and 2% melanin concentration increases monotonically from $f_{x}=0$ to ≈0.4/mm and then decreases slightly at higher spatial frequencies. For longer wavelengths, the maximal reflectance change is achieved at lower spatial frequencies. For instance, although the maximum reflectance change at λ=526  nm is observed at $f_{x}=0.4/\mathrm{mm}$, it shifts toward $f_{x}=0.2/\mathrm{mm}$ at λ=851  nm. Monitoring SFD reflectance at these intermediate spatial frequencies provides a maximal reflectance change for scattering changes within the papillary dermis.

Figures 3(b) and 3(c) provide the results for 5% and 10% epidermal melanin content, respectively. As seen, the overall spectral profile of the SFD reflectance change is similar to the results at 2% epidermal melanin content. Comparison of Figs. 3(a)–3(c) clearly shows that lower melanin concentrations provide larger reflectance changes while lowering the spatial frequency at which these reflectance changes appear. This can be more clearly seen in a contour map shown in Figs. 3(d)–3(f) which visualizes the predicted reflectance change $\Delta R_{d}$ versus spatial frequency $f_{x}$ on the x-axis and wavelength λ on the y-axis for epidermal melanin concentrations of 2%, 5%, and 10%, respectively.

The contour map for 2% melanin concentration, shown in Fig. 3(d), displays the highest intensity region, corresponding to the maximum reflectance change that occurs around λ=621  nm and $f_{x}\approx 0.3/\mathrm{mm}$. This suggests that this combination of wavelength and spatial frequency is most sensitive to changes in optical properties for lower melanin concentrations. As the melanin concentration increases to 5%, the peak region expands and shifts toward longer wavelengths, indicating that higher melanin concentrations require longer wavelengths and a broader range of spatial frequencies for optimal sensitivity. In the case of 10% melanin concentration, the peak region further expands and shifts, with wavelengths above 691 nm and spatial frequencies in the range of 0.22 to 0.32/mm showing the highest reflectance change. This highlights the significant impact of melanin on the choice of wavelength and spatial frequency for maximizing sensitivity to optical property changes. The overall decrease in the SFD reflectance change from Figs. 3(d)–3(f) is driven by the increased absorption by higher melanin concentrations.

**Fig. 4:**
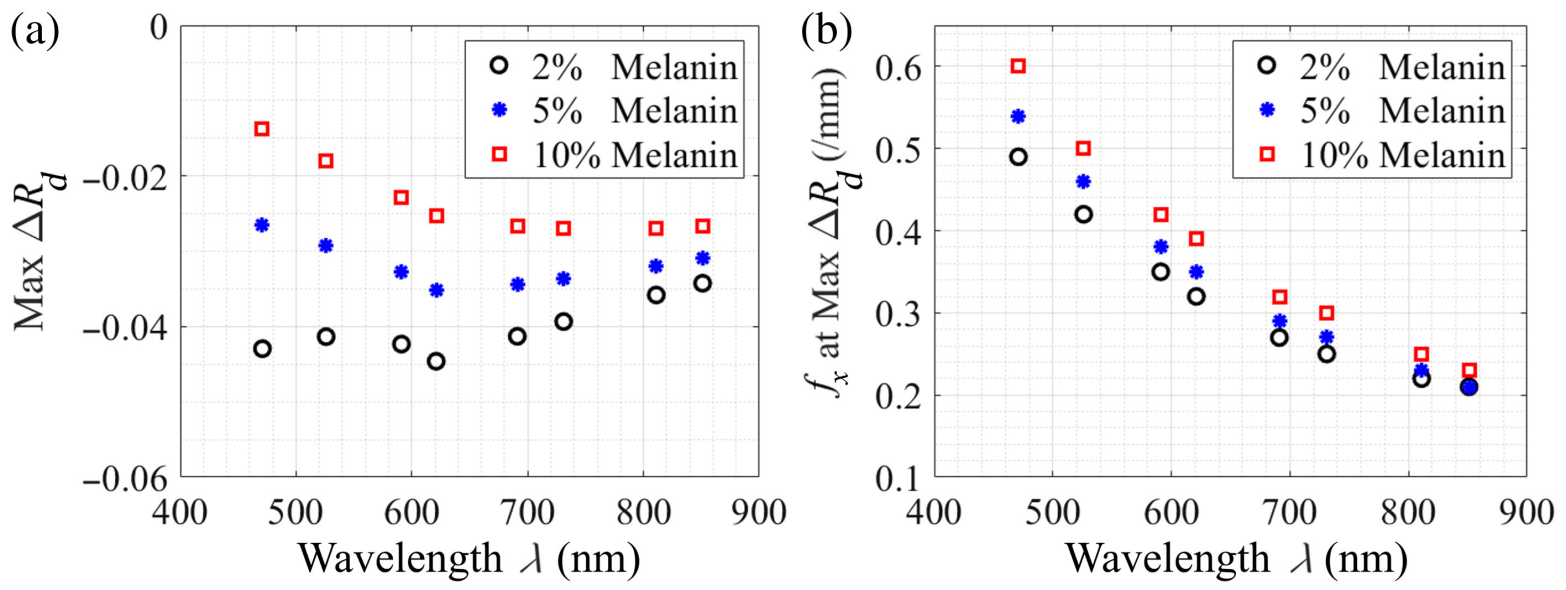
Wavelength dependence of (a) maximum reflectance changes, max ΔR, and (b) corresponding spatial frequency, $f_{x}$ at max ΔR for 2%, 5%, and 10% epidermal melanin concentrations.

Figures 4(a) and 4(b) provide the spectral variation of the maximum SFD reflectance change and the corresponding spatial frequencies across all melanin concentrations. As indicated in Fig. 3, the relationship between the maximum SFD reflectance change and wavelength is influenced by the melanin concentration. However, apart from the case of low (2%) melanin content, the greatest maximum reflectance change between the baseline and negative scattering perturbation case is consistently observed in the red/near-infrared spectral region of λ=621 to 851 nm, which provides the greatest reflectance change due to scattering changes in the papillary dermis for 5% and 10% epidermal melanin concentrations. Interestingly, the spatial frequency at which the largest reflectance change is observed reduces monotonically with increasing wavelength. Moreover, the variation in this spatial frequency across different melanin concentrations diminishes for longer wavelengths. Note that although the magnitude of the SFD reflectance will change with the magnitude of the scattering perturbation, the wavelength-spatial frequency combinations at which the maximal reflectance changes are achieved are minimally affected by the magnitude of the scattering perturbation given that the radiative transport equation is a linear equation relative to the optical coefficients of the material.

At first glance, the results shown in Fig. 4 may be surprising. For example, one might expect that the intrinsically smaller penetration depths in the blue/green spectral region as compared with the near-infrared (NIR) would result in a smaller spatial frequency that would provide the maximal reflectance change in response to a scattering perturbation. To gain insight into the effect of optical properties on the spatial frequency that provides a maximal reflectance change, we used conventional and perturbation MC simulations^8^^,^^56^^,^^57^ to determine an “optimal” spatial frequency $f_{x,\mathrm{opt}}$ that provides a maximal change in reflectance resulting from a scattering perturbation. For simplicity, and to provide generality, we considered a homogeneous medium and considered a range of optical properties, as expressed by the ratio of reduced scattering to absorption $\left(\mu_{s}^{\prime }/\mu_{a}\right)$ while retaining a constant transport mean free path $l^{*}=1\;\mathrm{mm}$. This allows us to define a dimensionless spatial frequency $f_{x}^{*}=f_{x}l^{*}$. This approach isolates the intrinsic relationship between optical properties and the sensitivity of reflectance to scattering changes by removing the effect of variations in the  transport mean free path.

**Fig. 5:**
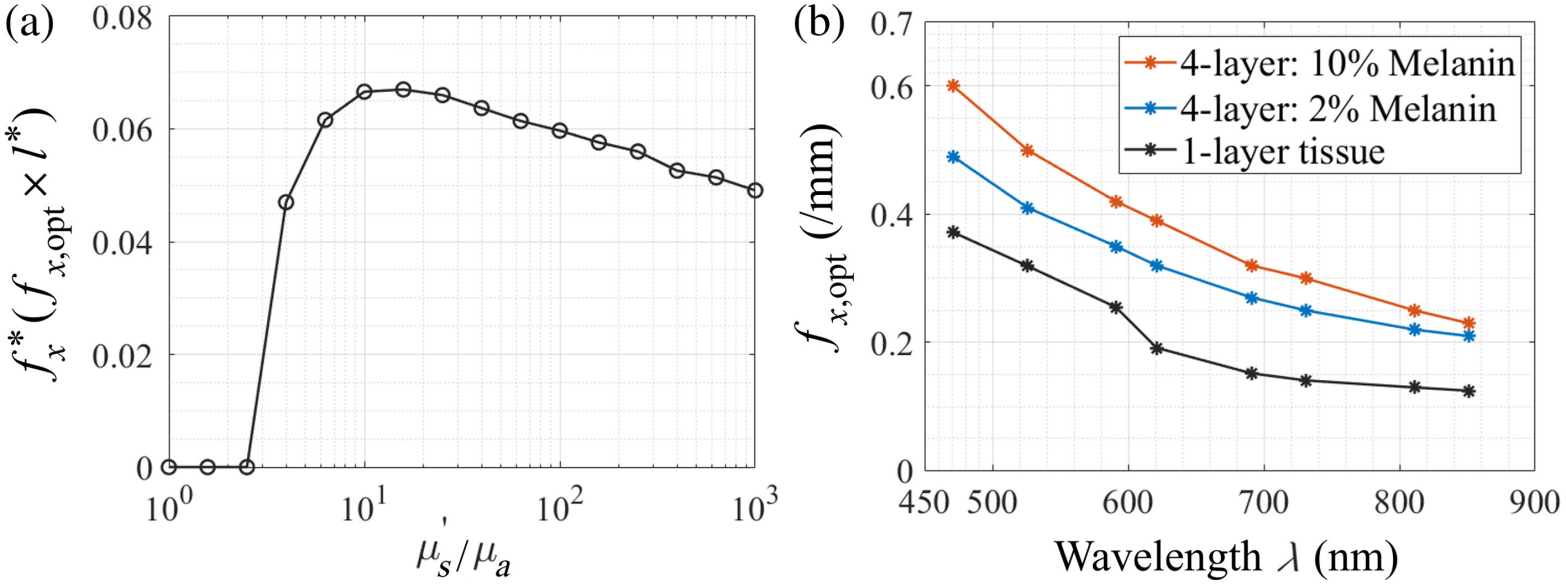
(a) Dimensionless “optimal” spatial frequency $f_{x}^{*}$ versus $\left(\mu_{s}^{\prime }/\mu_{a}\right)$. (b) Spectral dependence of the optimum spatial frequency $f_{x,\mathrm{opt}}$.

Figure 5(a) shows the results of these simulations that determine the dimensionless “optimum” spatial frequency, $f_{x}^{*}$ versus $\left(\mu_{s}^{\prime }/\mu_{a}\right)$. For context, the values of $\left(\mu_{s}^{\prime }/\mu_{a}\right)$ in the dermis lie in the range of 17 to 404 in the λ=470- to 850-nm spectral range considered in this paper. For smaller values of $(\mu_{s}^{\prime }/\mu_{a})≲3$, the dimensionless “optimal” spatial frequency $f^{*}$ is zero. This is due to the low amounts of remitted light due to significant absorption. As a result, uniform planar illumination is most sensitive to scattering changes in this regime of optical properties. For $(\mu_{s}^{\prime }/\mu_{a})\approx 3$ to 10, the increased availability of reflected light,^58^ combined with a more prominent reduction of path length offered by increased spatial frequency,^22^ leads to a rapid increase in the $f_{x}^{*}$ value that provides maximal scattering sensitivity. For $\left(\mu_{s}^{\prime }/\mu_{a}\right)$ ≳10, the $f_{x}^{*}$ value plateaus and then decreases slightly as scattering becomes more dominant. The slight decrease in $f_{x}^{*}$ with increasing $\left(\mu_{s}^{\prime }/\mu_{a}\right)$ occurs because the relative increase in scattering produces a more rapid decrease in reflectance with increasing spatial frequency. This, in turn, drives a reduction in the spatial frequency at which a maximal scattering sensitivity is attained.

Using the results from Fig. 5(a), we construct Fig. 5(b) which provides a comparison between the spectral variation of the optimum spatial frequency, $f_{x,\mathrm{opt}}$, as predicted by our homogeneous analysis using dermis optical properties with the spatial frequencies that provide the maximal change in SFD reflectance provided by our four-layer skin model for 2% and 10% epidermal melanin concentrations as shown in Fig. 4(b). The results of Fig. 5(b) show that the overall trends for the spectral variation of $f_{x,\mathrm{opt}}$ are preserved between the homogeneous and the more complex multilayer scenario. We see again a clear trend of a decreasing value of $f_{x,\mathrm{opt}}$ with increasing wavelength. Given that the dimensionless “optimal” spatial frequency $f_{x}^{*}$ does not vary considerably in the range $\left(\mu_{s}^{\prime }/\mu_{a}\right)$ values exhibited by the dermis in this spectral range, this trend is clearly driven by the roughly 2.6-fold increase in the transport mean free path at λ=851  nm as compared with λ=471  nm. When comparing the results of the homogeneous analysis with the predictions of the four-layer model, we see uniformly higher optimum spatial frequencies. This is driven by two factors: (a) the highly absorbing epidermal layer which attenuates more light at lower frequencies that serves to shift the optimal spatial frequency to higher values and (b) the fact that the scattering perturbation occurs only in the superficial dermis as opposed to the entire tissue volume as is the case in the one-layer (homogeneous) tissue model. We discuss the complexities of the multi-layer scenario in Sec. 4.

## 4 Discussion

Many studies have investigated the promise of leveraging the well-established depth sensitivity of multi-frequency SFDI^22^ to determine the optical properties and tissue composition of layered tissues.^9^^,^^10^^,^^13^^,^^39^^,^^41^^,^^59^^,^^60^ These studies explicitly consider the inverse problem utilizing a variety of approaches ranging from the use of Monte Carlo lookup tables, analytical light transport models,^10^^,^^23^ machine learning,^41^ and heuristic multi-wavelength layered tissue models.^13^^,^^60^ Our SLIM approach bypasses entirely the process of explicit recovery of tissue optical/physiological properties. Instead, SLIM identifies wavelength–spatial frequency pairs that are optimally suited for the detection of subsurface scattering changes. Below, we discuss various considerations for the determination of optimal measurement parameters to provide maximal sensitivity to these changes.

### 4.1 Wavelength and Spatial Frequency Selection

Our results in Fig. 3 show that an embedded scattering perturbation produces a non-monotonic change in measured reflectance change versus spatial frequency. This results in an intermediate spatial frequency at which the reflectance change is maximized. In the case of skin, our results in Fig. 4(a) show that for skin with low to moderate epidermal melanin content, maximal reflectance changes are observed in the λ=471- to 621-nm spectral region. For low to moderate melanin content, we show that the largest reflectance change occurs at λ=621  nm [Fig. 4(a)] using a spatial frequency of $f_{x}\approx 0.32$ to 0.35/mm [Fig. 4(b)]. Moreover, in the λ=471- to 621-nm spectral region, the maximum reflectance change is very sensitive to the epidermal melanin content.

By contrast, for highly pigmented skin (10% epidermal melanin), SFD measurements at λ≳700  nm provide the largest reflectance change, as shown in Fig. 4(a). Moreover, as shown in Fig. 4(b), the use of longer wavelengths provides the maximal reflectance change at lower spatial frequencies, which may be practically advantageous due to a better instrument signal-to-noise ratio.^11^ Specifically, Fig. 4(b) identifies that SFD measurements taken at λ=811 or 851 nm paired with a spatial frequency of $f_{x}\approx 0.25/\mathrm{mm}$ provide the largest reflectance change when investigating highly pigmented subjects.

An important advantage of making measurements at λ≳700  nm, as opposed to λ≲621  nm, is that the observed reflectance change is minimally dependent on melanin content. As such, if a single set of measurement parameters is desired to capture reflectance contrast across skin samples with varying pigmentation levels, the results shown in Fig. 4 suggest that the $\lambda \text{-}f_{x}$ pairing of λ=851  nm and $f_{x}\approx 0.22/\mathrm{mm}$ would provide a relatively consistent reflectance change.

### 4.2 Origins of Reflectance Changes and Penetration Depth Analysis

To better understand the origins of the reflectance change produced by a decrease in papillary dermis scattering, we performed a penetration depth analysis for both the baseline and perturbed cases. We utilized the penetration depth analysis method described in a previous work from our group,^22^ which involves calculating the depth-dependent probability distribution of photon packet visitation and detection ($p_{V\cap D}$). The maximum penetration depth distribution, $p_{z_{\max}}(z)$, is derived by tallying the contribution of each detected photon packet to its maximum depth visited. This allows for the generation of a density function that tallies the weight of each detected photon at the z location representing the maximum depth visited by that photon’s trajectory in the tissue. Such a distribution ensures that each photon’s weight is tallied only once such that the integral of the $p_{z_{\max}}(z)$ density function recovers the measured reflectance. Thus, this density function identifies the depths from which the measured reflectance originates and provides quantitative insight into the sampling depth for each spatial frequency.

**Fig. 6:**
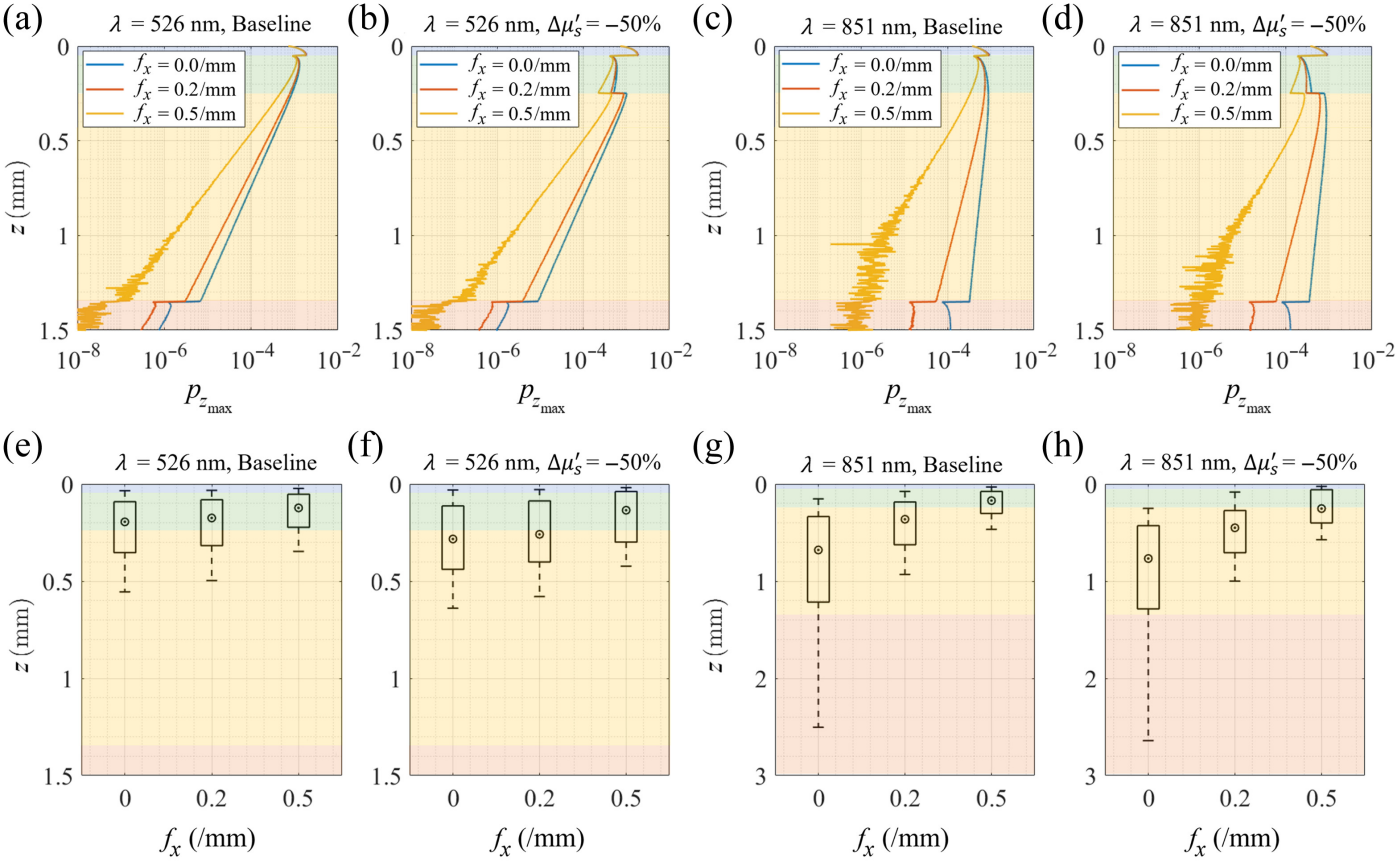
Plots of $p_{z_{\max}}(z)$ for (a) and (b) λ=526  nm and (c) and (d) λ=851  nm before and after a 50% reduction in scattering within the papillary dermis, respectively, at $f_{x}=0,0.2$, and 0.5/mm. Bar plots providing sampling depth statistics at (e) and (f) λ=526  nm and (g) and (h) λ=851  nm before and after scattering a 50% reduction in scattering within the papillary dermis, respectively. The median depth ($d_{50}$) is represented by a circled dot, intervals of 25 to 75% ($d_{25}$ to $d_{75}$) by a rectangle, and 10 to 90% ($d_{10}$ to $d_{90}$) by a capped line at the same spatial frequencies as the $P_{z_{\max}}$ distributions. The shading of the plots is color-coded as follows: blue for the epidermis, green for the papillary dermis, yellow for the reticular dermis, and red for the subcutaneous tissue. All plots are for 2% epidermal melanin concentration. Results for 5% and 10% melanin concentrations are shown in Figs. S1 and S2 in the Supplementary Material, respectively.

Figures 6(a)–6(b) and 6(c)–6(d) plot the $p_{z_{\max}}$ density function and associated depth statistics of the detected photons at λ=526 and 851 nm, respectively, with 2% epidermal melanin concentration for baseline [Figs. 6(a) and 6(c)] and perturbed [Figs. 6(b) and 6(d)] cases. Reduction of the papillary dermis scattering coefficient produces a more gradual decay of the $p_{z_{\max}}$ distribution within the papillary dermis and a higher total penetration depth for each of the perturbed cases.

To quantify these changes in penetration depth, we utilize the cumulative distribution $P_{z_{\max}}(z\leq d)=\int_{0}^{d}p_{z_{\max}}(z)dz$, which represents the portion of detected photons with maximum penetration depths not exceeding depth d. By normalizing this distribution with respect to the total diffuse reflectance $R_{d}$, we can define a metric $d_{m}$, where m represents a percentage of detected light. This metric is determined by^22^

$$m=100\times [P_{z_{\max}}(z\leq d_{m})/R_{d}].$$ (5)

Figures 6(e)–6(h) depicts computed depths $d_{10},d_{25},d_{50},d_{75}$, and $d_{90}$, corresponding to the tissue depths that enclose the photon packet trajectories responsible for m values corresponding to 10%, 25%, 50%, 75%, and 90% of the detected reflectance, respectively. These results show a stronger attenuation of the photon visitation probability at higher spatial frequencies and shorter wavelengths, indicative of lower penetration depths for these cases.

The $p_{z_{\max}}$ distribution plots show that the scattering coefficient reduction within the papillary dermis in the perturbed case results in increased penetration depths for both wavelengths at all spatial frequencies. In addition, the penetration depth is smallest at the highest spatial frequency. In all cases, the median depth lies within the papillary dermis at $f_{x}=0.5/\mathrm{mm}$. Moreover, at longer wavelengths, light travels deeper into the tissue due to the lower tissue scattering and absorption coefficients. For instance, at λ=851  nm and $f_{x}=0/\mathrm{mm}$, 75% of the detected reflectance emanates from depths no larger than 1.3 mm whereas for λ=526  nm, 75% of the detected reflectance originates from depths no larger than 0.5 mm at $f_{x}=0/\mathrm{mm}$.

The wavelength–spatial frequency pair that provides the largest change in SFD reflectance is linked to the change in the $p_{z_{\max}}$ distribution caused by the scattering perturbation. The Monte Carlo results shown in Fig. 4 demonstrate that for low to moderate epidermal melanin concentrations, mid-range wavelengths (λ=621  nm) and higher spatial frequencies ($f_{x}=0.32$ to 0.35/mm) provide the largest change in reflectance resulting from scattering reductions within the papillary dermis. By contrast, for high epidermal melanin concentration, long wavelengths (811 to 851 nm) and lower $f_{x}$ (0.25/mm) provide the largest reflectance change. The $p_{z_{\max}}$ distribution and sampling depth plots for high melanin concentration are provided in the Supplementary Material.

### 4.3 Origins of Reflectance Changes and the Maximum Depth Density Function $p_{z_{\max}}(z)$

**Fig. 7:**
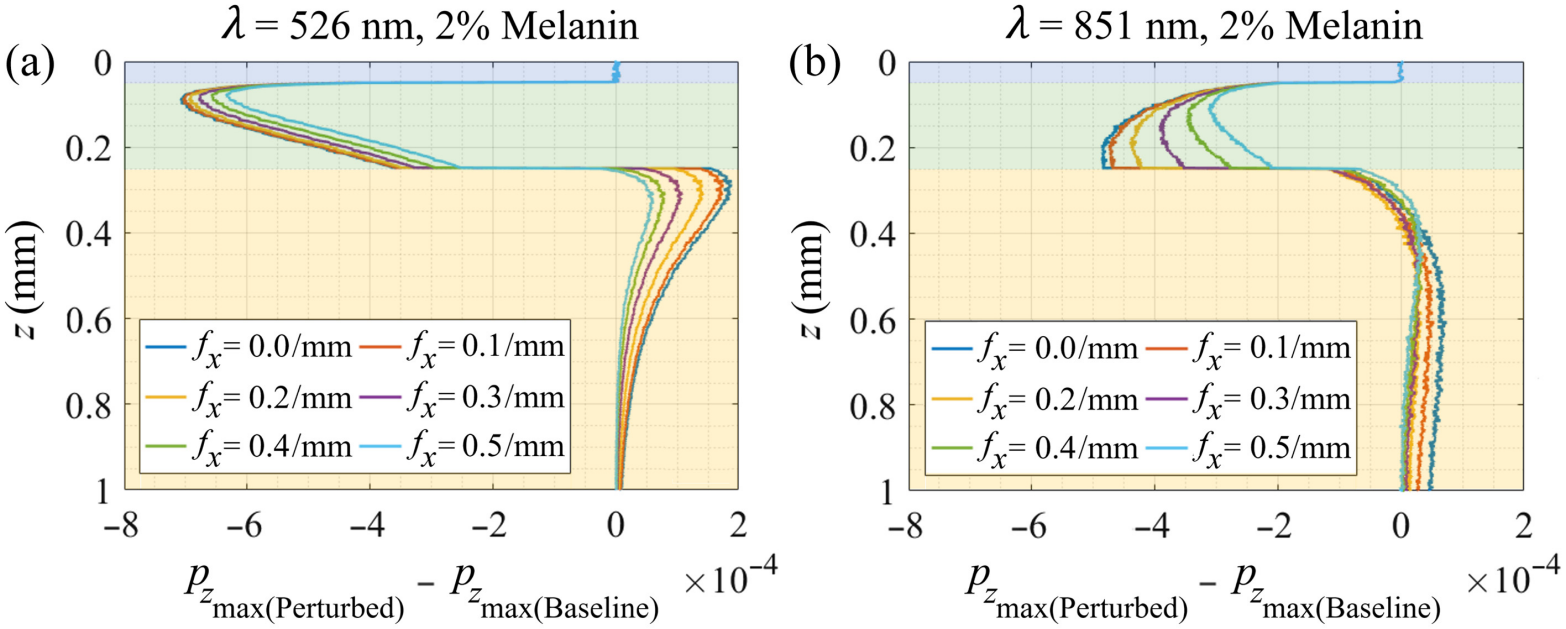
Maximum penetration depth distribution difference before and after scattering perturbation at different spatial frequencies for (a) 526 nm and (b) 851 nm at 2% melanin concentration. Plots are color-coded as follows: blue for the epidermis, green for the papillary dermis, and yellow for the reticular dermis. Results for 5% and 10% melanin concentrations are shown in Figs. S3 and S4 in the Supplementary Material, respectively.

To better understand the effect of the localized scattering change on the measured reflectance, the difference in the $p_{z_{\max}}(z)$ distribution between the perturbed case and the baseline case is shown in Fig. 7. Figure 7(a) provides this result for λ=526  nm and shows that the reduction in scattering in the papillary dermis reduces the reflectance coming from this layer. This also results in increased photon penetration that increases the reflectance contribution from the underlying reticular dermis. At low spatial frequencies, the signal is sensitive to both the reflectance decrease from the papillary dermis and reflectance increase from the reticular dermis. However, as $f_{x}$ increases, the reflectance increase from the reticular dermis is greatly diminished, whereas the reflectance decrease produced by the scattering reduction of the papillary dermis is largely preserved. As a result, we see a maximal net reduction of reflectance around $f_{x}\approx 0.35/\mathrm{mm}$, which is consistent with the findings shown in Figs. 3–6.

In Fig. 7(b), we show the difference in the $p_{z_{\max}}$ distribution between the baseline and perturbed case for λ=851  nm. A similar dynamic is at play where the reduced backscatter from the papillary dermis due to the reduction in scattering is offset somewhat by increased backscatter from the reticular dermis. However, as optical penetration in tissue is much greater in the red-NIR spectral regions, increases in the spatial frequency from $f_{x}=0/\mathrm{mm}$ have a greater impact on reducing the backscatter from the reticular dermis and minimal impact in reducing the backscatter from the papillary dermis. As a result, we see large reductions in the reflectance emanating from the reticular dermis until $f_{x}\approx 0.2/\mathrm{mm}$. However, further increases in the spatial frequency beyond $f_{x}\approx 0.2/\mathrm{mm}$ begin to limit the penetration of the incident light in the papillary dermis which reduces the reflectance reduction offered by the scattering perturbation in the papillary dermis. This occurs at a lower spatial frequency because the transport mean free path in the dermis at λ=851  nm is more than double that at λ=526  nm (0.46 versus 0.21 mm). This results in a lower spatial frequency ($f_{x}\approx 0.2/\mathrm{mm}$) at which we are sensitive to scattering changes and provides a maximal difference between baseline and perturbed cases for λ=851  nm.

**Fig. 8:**
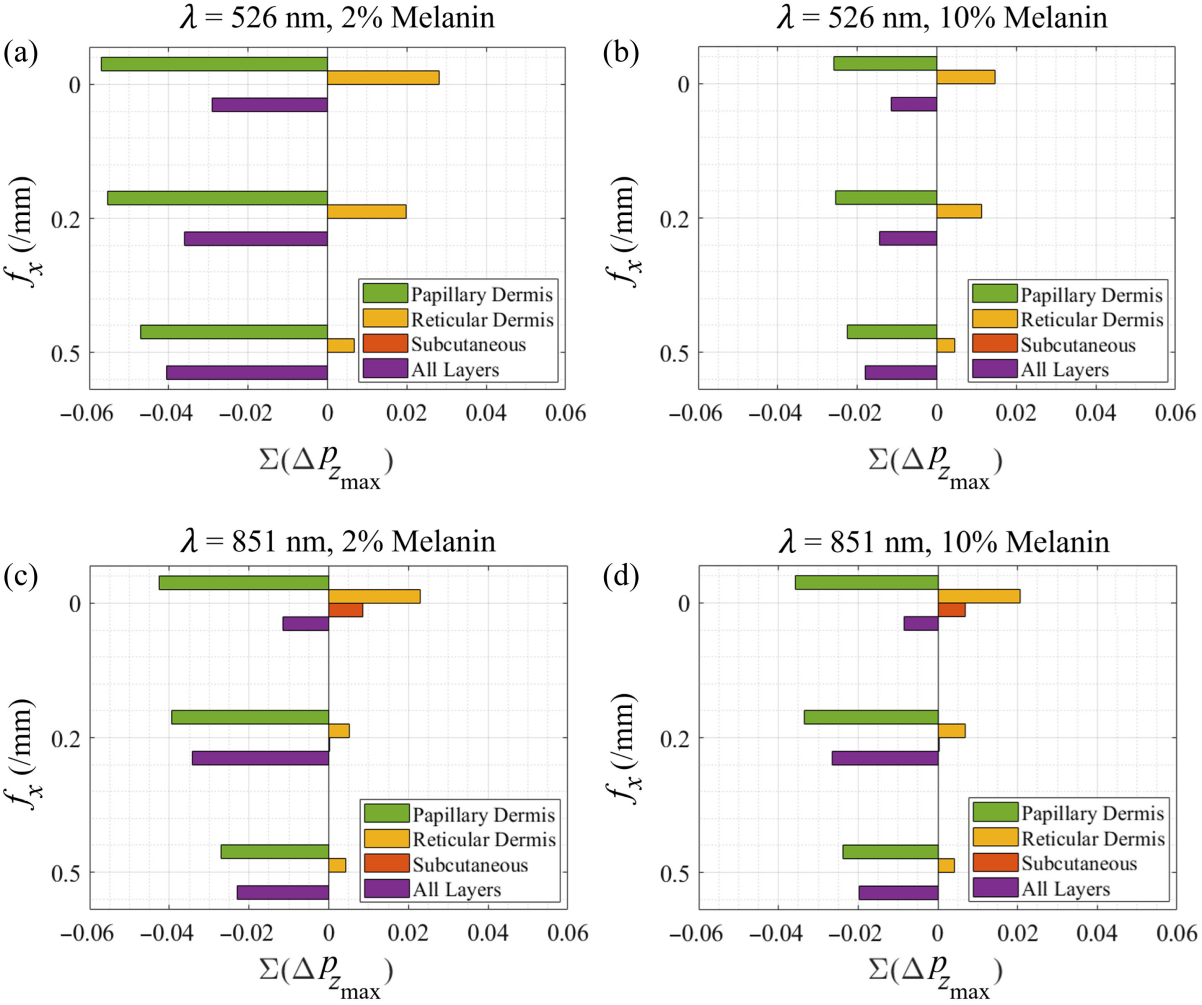
Individual layer contributions to the total reflectance change at spatial frequencies: $f_{x}=0$, 0.2, and 0.5/mm for λ=526  nm with (a) 2% and (b) 10% melanin concentration and λ=851  nm with (c) 2% and (d) 10% melanin concentration. Due to negligible contribution, the epidermis was excluded from the plots. The subcutaneous tissue layer’s contribution is also negligible at shorter wavelengths (λ=526  nm) due to limited light penetration. Results for 5% melanin concentration are shown in Fig. S5 in the Supplementary Material.

These features can be better appreciated by displaying the layer-specific contributions to the total reflectance change resulting from the scattering perturbation in the papillary dermis at λ=526 and 851 nm for cases of low (2%) and high (10%) melanin concentration as shown in Fig. 8. These results show that in all cases the individual sublayer contributions to the total reflectance change decreases as spatial frequency increases. The results for λ=526  nm are shown for low and high melanin concentrations in Figs. 8(a) and 8(b). These show that increases in spatial frequency have the effect of reducing the reflectance change from the reticular dermis more so than the papillary dermis. As a result, the maximum total reflectance change occurs at the highest spatial frequencies of $f_{x}=0.42$ to 0.5/mm depending on epidermal melanin content as shown in Fig. 4. On the other hand, for λ=851  nm, shown in Figs. 8(c) and 8(d), there is a specific point at $f_{x\;}\approx 0.2/\mathrm{mm}$ where further increases in the spatial frequency do not result in additional reductions in backscattering from the reticular dermis while also decreasing the reflectance provided by the papillary dermis. As a result, the maximal change in SFD reflectance at $f_{x}=0.21$ to 0.23/mm depending on epidermal melanin content as shown in Fig. 4.

Comparison of Figs. 8(a) and 8(b) with Figs. 8(c) and 8(d) reveals that the spatial frequency at which we see the largest change in reflectance due to the reduction in scattering in the papillary dermis layer shifts toward lower spatial frequencies with increasing wavelength. This is seen directly in Fig. 4.

The reflectance changes for the case of low (2%) melanin concentration are highest for mid-range wavelengths. Specifically, measurements at λ=621  nm and $f_{x}\approx 0.33/\mathrm{mm}$ represent the best wavelength and spatial frequency pair offering the highest sensitivity to the embedded reductions in scattering coefficient in the low melanin concentration skin model. In the case of high (10%) epidermal melanin content, wavelengths longer than 691 nm provide the highest reflectance measurement contrast. For these subjects, the wavelength–spatial frequency combination of λ=811 or 851 nm and $f_{x}\approx 0.20$ to 0.25/mm provide the largest reflectance change for scattering reductions in the papillary dermis. Moreover, if one desires a single wavelength–spatial frequency pair that optimizes for reflectance changes across all pigmentation levels, our results suggest the use of λ=851  nm and $f_{x}\approx 0.22/\mathrm{mm}$. This combination minimizes the variation in observed reflectance changes across melanin concentrations ranging from 2% to 10%.

Collectively, our results demonstrate spatial frequency, wavelength, and melanin concentration dependence of reflectance in a multi-layer skin tissue model. The introduction of a reduction in the scattering coefficient within an embedded tissue layer (papillary dermis) to simulate a disease pathology leads to a consistent reduction in reflectance across all spatial frequencies and wavelengths as compared with the baseline simulation but with a maximal reflectance change occurring at an intermediate spatial frequency. It is important to note that the optimal wavelength–spatial frequency pairings are specific to parameters within the skin model. Specifically, these pairings are sensitive to the magnitude of the scattering changes and both the thickness and depth of the subsurface layer in which the scattering changes occur. Nevertheless, this approach of identifying the layer-specific contributions to the measured reflectance, and its functional dependence on both wavelength and spatial frequency, enables SLIM to identify the wavelength–spatial frequency pair best suited to detect changes in subsurface scattering within a tissue subvolume of interest.

## 5 Summary and Conclusion

SLIM represents a simplified approach to utilizing the raw SFDI reflectance measurements alone to detect subsurface scattering changes in tissues, that bypasses entirely the inversion to optical/physiological properties. The technique has its basis in specifying particular $\lambda \text{-}f_{x}$ pairs that provide a maximal reflectance change.

Our analysis highlights the complex interplay among spatial frequency, wavelength, layered tissue architecture, and melanin concentration that influences changes in reflectance and sampling depth in the skin. Within the context of this model, our results show that the $\lambda \text{-}f_{x}$ pair of λ=621  nm and $f_{x}=0.33/\mathrm{mm}$ represents the largest reflectance change for detecting subsurface scattering reductions in skin samples with low epidermal melanin concentration (2%). In skin samples with high levels of epidermal melanin (10%), this optimal $\lambda \text{-}f_{x}$ pair changes to λ=811 or 851 nm and $f_{x}=0.25/\mathrm{mm}$. Finally, if one desires to identify a single $\lambda \text{-}f_{x}$ pair that optimizes reflectance changes across samples with varying concentrations of epidermal melanin, our results suggest a measurement configuration using λ=851  nm and $f_{x}\approx 0.22/\mathrm{mm}$ within the context of our model. We recognize that other elements of tissue structure and composition may change without a scattering change in the targeted tissue volume which may cause reflectance changes at the designated $\lambda \text{-}f_{x}$ pair resulting in a “false positive” when using the SLIM approach. In part 2 of this paper published in *Biophotonics Discovery*,^1^ we show that in the case of systemic sclerosis, this appears not to be the case and a single reflectance measurement at the SLIM-specified $\lambda \text{-}f_{x}$ pair provides sufficient power to discriminate diseased from normal patients. It is important to note that changes in scattering within a given tissue layer or changes in the thickness of such a layer provide a unique non-monotonic change in variation of measured reflectance with spatial frequency. Nonetheless, if one wishes to better exclude false positives, a strategy of making measurements at multiple spatial frequencies would have better discriminatory power at the expense of measurement time and complexity.

In conclusion, by carefully selecting measurement parameters, SLIM can provide sensitivity to subsurface scattering changes in layered tissues, offering potential improvements in non-invasive diagnostics without explicit consideration of the complex layered tissue inverse problem. In practice, we imagine using the SLIM approach to image a patient population, which would consist of both “healthy” and “diseased” subjects, which can be compared with each other. Alternatively, for a given patient, SLIM could be used to track properties longitudinally, to monitor disease progression, or response to treatment. The application of SLIM to a disease state is elucidated further in part 2 of this paper published in *Biophotonics Discovery*.^1^

## Supplementary Material

10.1117/1.JBO.30.6.065001.s01

## Data Availability

The open-source MCCL application, available at https://virtualphotonics.org/software, was utilized to generate all the data for this study. The complete dataset for this study is sizeable and derived from Monte Carlo simulations representing over 3000 distinct combinations of wavelength, spatial frequency, papillary dermis scattering, and melanin concentration. Simulation results for cases of interest can be provided upon request.
