# Supplementary material for: Structured light imaging mesoscopy: detection of embedded morphological changes in superficial tissues
Source: J Biomed Opt. 2025 Jun 18;30(6):065001. doi: 10.1117/1.JBO.30.6.065001 (PMC12175002; doi:10.1117/1.JBO.30.6.065001)
Supplement: Supplementary file 1 [file JBO_030_065001_SD001.pdf]

Supplementary Materials for

"Structured Light Imaging Mesoscopy: Detection of embedded morphological changes in superficial tissues" by Mahsa Parsanasab, Aarohi Mahesh Mehendale, Kavon Karrobi, Darren Roblyer, and Vasan Venugopalan

Optical Properties of the 4-Layer Model

Table S1. Absorption Coefficient Values (/mm)

Layers \ Wavelength	471nm	526nm	591nm	621nm	691nm	731nm	811nm	851nm
Epidermis (No Melanin)	0.000425	0.000117	0.000079	0.000090	0.000113	0.000373	0.000394	0.000804
Papillary Dermis	0.311598	0.355556	0.162893	0.021515	0.007056	0.007084	0.010394	0.013647
Reticular Dermis	0.311598	0.355556	0.162893	0.021515	0.007056	0.007084	0.010394	0.013647
Subcutaneous Tissue	1.090780	1.244452	0.569843	0.074840	0.023895	0.021509	0.032910	0.040173

Table S2. Baseline Reduced Scattering Coefficient Values (/mm)

Layers \ Wavelength	471nm	526nm	591nm	621nm	691nm	731nm	811nm	851nm
Epidermis	7.804572	6.537840	5.499077	5.131983	4.462107	4.166001	3.703192	3.519963
Papillary Dermis	5.334748	4.382894	3.611039	3.340689	2.851527	2.637381	2.305911	2.175989
Reticular Dermis	5.334748	4.382894	3.611039	3.340689	2.851527	2.637381	2.305911	2.175989
Subcutaneous Tissue	1.732721	1.512741	1.335478	1.272420	1.154762	1.100830	1.012454	0.975522

Table S3. Epidermis Absorption Coefficient Values with Different Melanin Concentrations(/mm)

Melanin (%) \ Wavelength	471nm	526nm	591nm	621nm	691nm	731nm	811nm	851nm
2	1.696192	1.154762	0.769838	0.648008	0.446895	0.367695	0.256304	0.217237
5	2.544075	1.732085	1.154717	0.971967	1.117067	0.918677	0.640168	0.541887
10	3.391959	2.309407	1.539596	1.295926	2.234021	1.836981	1.279942	1.082971

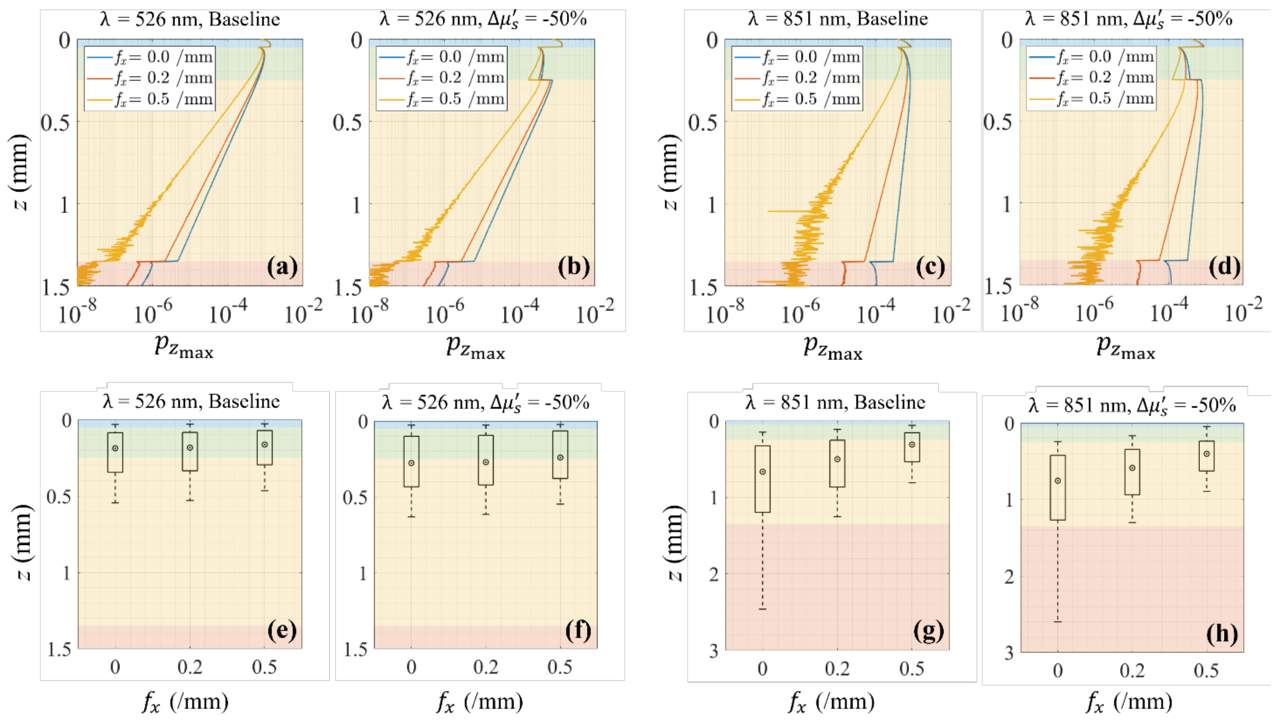

Figure S1: Plots of $P_{z_{\max}}(z)$ for (a), (b) λ = 526 and (c), (d) λ = 851nm before and after a 50% reduction in scattering within the papillary dermis, respectively, at f_x= 0, 0.2 and 0.5 /mm. Bar plots providing sampling depth statistics at (e), (f) λ = 526 and (g), (h) λ = 851nm before and after scattering a 50% reduction in scattering within the papillary dermis, respectively. The median depth (d_{50}) is represented by a circled dot, intervals of [25 to 75] % (d_{25} to d_{75}) by a rectangle, and [10 to 90] % (d_{10} to d_{90}) by a capped line at the same spatial frequencies as the $P_{z_{\max}}$ distributions. Plots are color-coded as follows: blue for epidermis, green for papillary dermis, yellow for reticular dermis, and red for subcutaneous tissue. All plots are for **5% epidermal melanin** concentration.

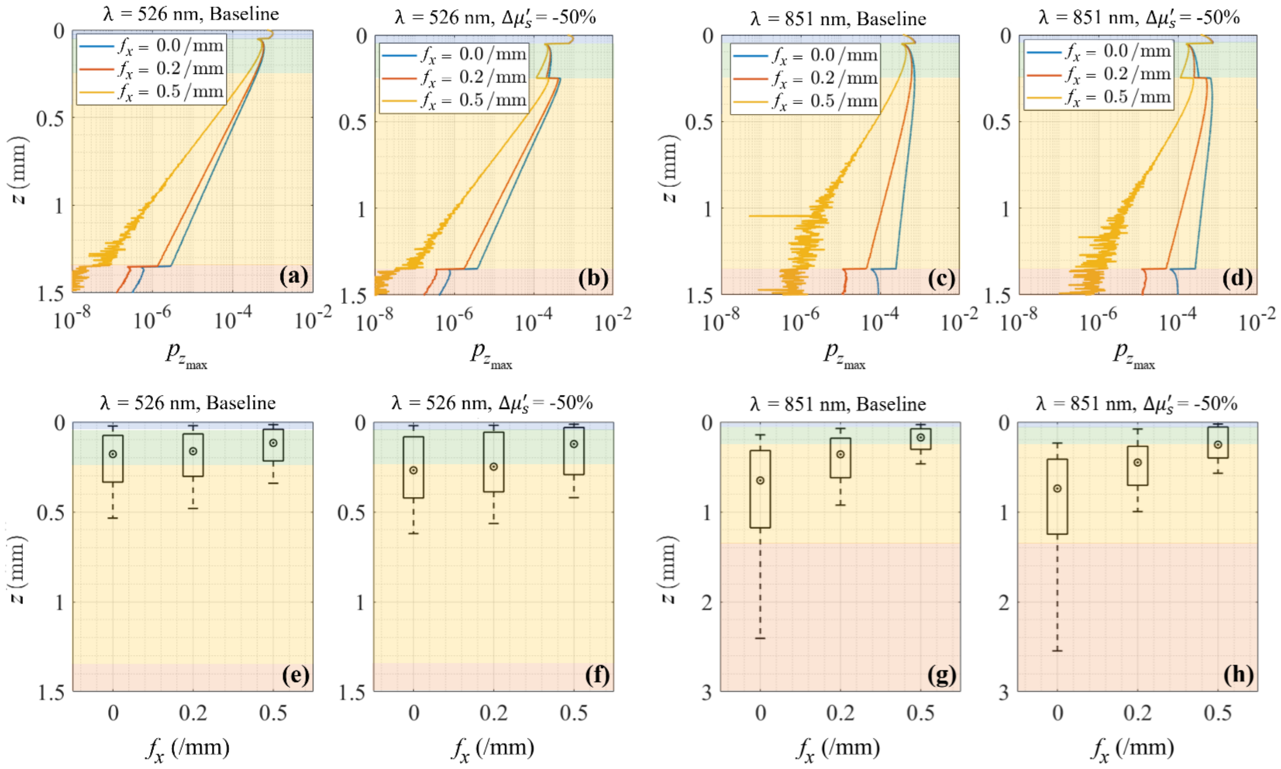

Figure S2: Plots of $P_{z_{max}}(z)$ for (a), (b) λ = 526 and (c), (d) λ = 851nm before and after a 50% reduction in scattering within the papillary dermis, respectively, at f_x= 0, 0.2 and 0.5 /mm. Bar plots providing sampling depth statistics at (e), (f) λ = 526 and (g), (h) λ = 851nm before and after scattering a 50% reduction in scattering within the papillary dermis, respectively. The median depth (d_{50}) is represented by a circled dot, intervals of [25 to 75] % (d_{25} to d_{75}) by a rectangle, and [10 to 90] % (d_{10} to d_{90}) by a capped line at the same spatial frequencies as the $P_{z_{max}}$ distributions. Plots are color-coded as follows: blue for epidermis, green for papillary dermis, yellow for reticular dermis, and red for subcutaneous tissue. All plots are for **10% epidermal melanin** concentration.

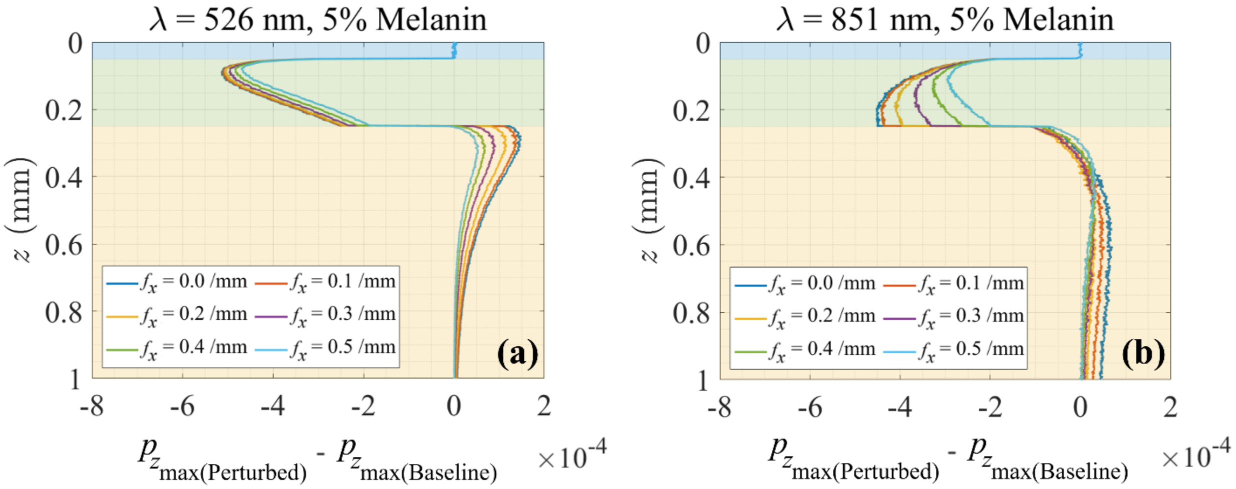

Figure S3: Maximum Penetration depth distribution difference before and after scattering perturbation at different spatial frequencies for (a) 526 nm and (b) 851 nm at **5% epidermal melanin** concentration. Plots are color-coded as follows: blue for epidermis, green for papillary dermis, and yellow for reticular dermis.

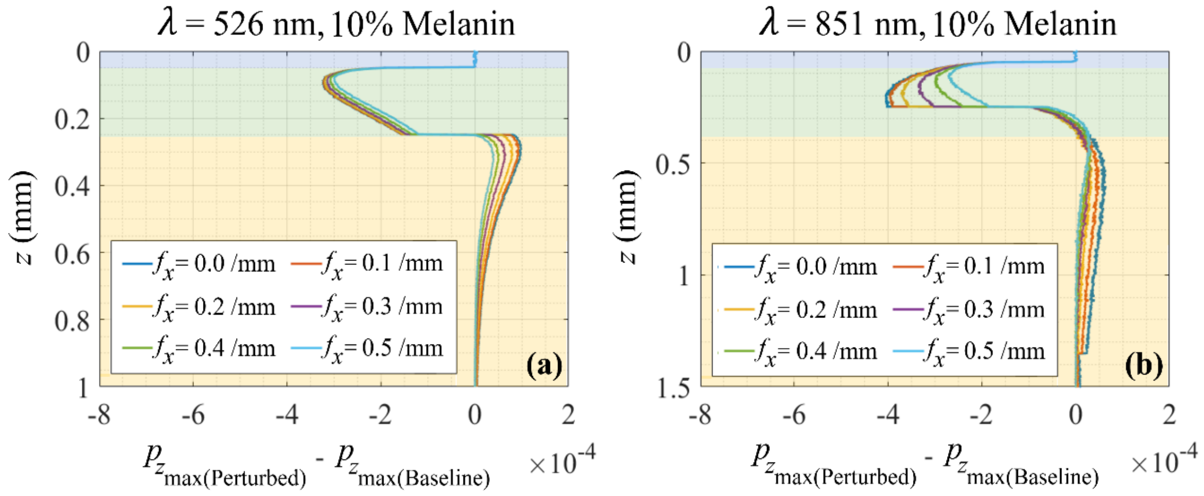

Figure S4: Maximum Penetration depth distribution difference before and after scattering perturbation at different spatial frequencies for (a) 526 nm and (b) 851 nm at **10% epidermal melanin** concentration. Plots are color-coded as follows: blue for epidermis, green for papillary dermis, and yellow for reticular dermis.

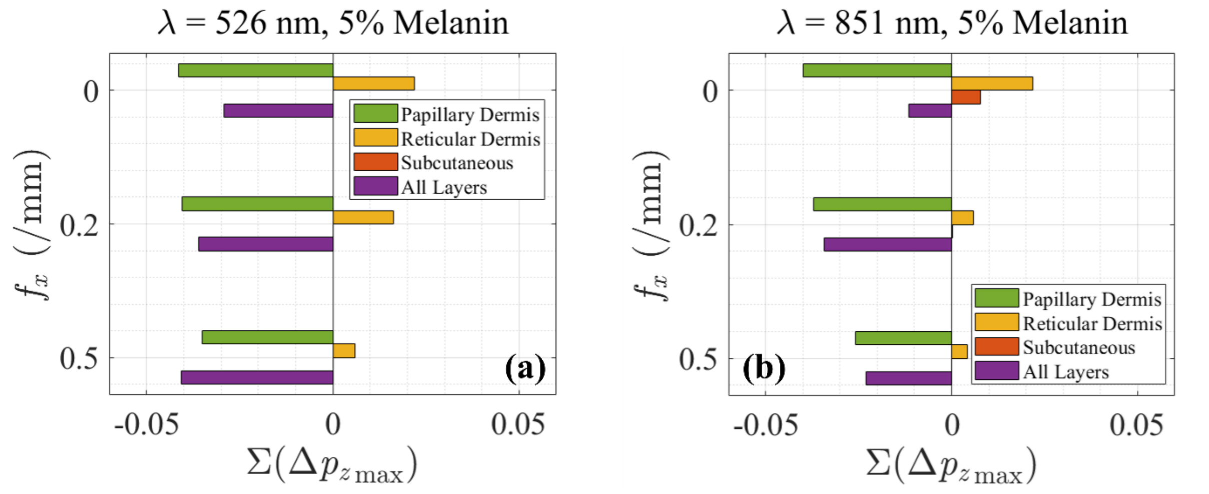

Figure S5: Individual layer contributions to the total reflectance change at spatial frequencies: f_x=0, 0.2, 0.5 /mm for (a) λ = 526 nm and (b) λ = 851 nm with **5% epidermal melanin** concentration. Due to negligible contribution, the epidermis was excluded from the plots. The subcutaneous tissue layer's contribution is also negligible at shorter wavelengths (λ = 526 nm) due to limited light penetration.
